# Correcting for Population Structure and Kinship Using the Linear Mixed Model: Theory and Extensions

**DOI:** 10.1371/journal.pone.0075707

**Published:** 2013-10-28

**Authors:** Gabriel E. Hoffman

**Affiliations:** Department of Biological Statistics and Computational Biology, Cornell University, Ithaca, New York, United States of America; Universite de Montreal, Canada

## Abstract

Population structure and kinship are widespread confounding factors in genome-wide association studies (GWAS). It has been standard practice to include principal components of the genotypes in a regression model in order to account for population structure. More recently, the linear mixed model (LMM) has emerged as a powerful method for simultaneously accounting for population structure and kinship. The statistical theory underlying the differences in empirical performance between modeling principal components as fixed versus random effects has not been thoroughly examined. We undertake an analysis to formalize the relationship between these widely used methods and elucidate the statistical properties of each. Moreover, we introduce a new statistic, effective degrees of freedom, that serves as a metric of model complexity and a novel low rank linear mixed model (LRLMM) to learn the dimensionality of the correction for population structure and kinship, and we assess its performance through simulations. A comparison of the results of LRLMM and a standard LMM analysis applied to GWAS data from the Multi-Ethnic Study of Atherosclerosis (MESA) illustrates how our theoretical results translate into empirical properties of the mixed model. Finally, the analysis demonstrates the ability of the LRLMM to substantially boost the strength of an association for HDL cholesterol in Europeans.

## Introduction

Population structure and kinship represent genetic relatedness between samples at different scales, and are widespread confounding factors in genome-wide association studies (GWAS) that can decrease power and increase the false positive rate of tests of association [1]. As a result, it is common practice to infer population structure and kinship based on genome-wide SNP data and to exclude problematic individuals or account for these effects in the test of association [1]. Principal components analysis (PCA) is widely used to detect population structure [2]. The inferred principal components capturing the genetic ancestry of each individual are often included as fixed effects in a regression-based test of association in order to account for population structure [3], [1]. More recently, a linear mixed model (LMM) that considers the genome-wide similarity between all pairs of individuals was proposed to account for population structure, known kinship as well as cryptic relatedness [4], [5], and recent technical advances have made such models tractable for very large datasets [4], [6], [7], [8], [9], [10], [11], [12].

While simple tests of association assume statistical independence between individuals, population structure and kinship indicate covariance between individuals based on the genetic similarity between individuals and the heritability of the phenotype [4]. Since it is well established that ignoring this covariance in a test of association produces deflated p-values that do not follow a uniform distribution under the null [13], it is common to apply a LMM or include principal components as fixed effects in order to model the dependence structure [1]. Both approaches model this covariance between individuals, and both can be stated as regressing the phenotype on principal components of the genotype matrix [14], [15], [16] so that the LMM essentially includes principal components as a random effect rather than a fixed effect. While the top principal components capture population structure, explicitly modeling the pairwise relatedness between all individuals captures both population structure and kinship [4], [1], [17], [18]. Thus much recent attention has focused on the LMM since it shows better empirical performance in modeling the dependence structure of GWAS datasets [4], [1], [17], [18].

Motivated by the empirical differences between the LMM and including principal components as fixed effects, we describe a unified framework that connects these models. This framework facilitates a statistical examination of the methods' differing frequentist vs. Bayesian interpretations, their differing approaches to inference and how these differences drive their empirical properties. We next introduce a summary statistic, the effective degrees of freedom, that measures overall model complexity and the influence of each principal component on the fit of the LMM. Leveraging the unified framework and the effective degrees of freedom, we propose a novel method, the low rank linear mixed model (LRLMM) using the algorithm of Lippert, et al. [6], that learns the dimensionality of the correction for population structure and kinship.

## Methods

### Modeling principal components as fixed versus random effects

Considering the matrix of genotype data 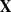 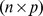 for 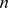 individuals and 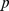 genetic markers, where entry 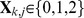 represents the number of copies of the minor allele that individual 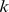 has of marker 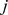, the singular value decomposition underlying principal components analysis (PCA) has the form
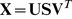
(1)so that the first 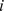 principal components are the first 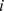 columns of 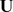 (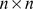), 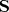 (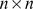) is diagonal so that 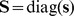 where 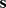 contains singular values corresponding to each principal component, 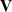 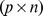 stores the loadings on each marker, and each marker in 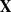 has been mean centered and scaled [2]. Including the first 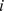 principal components as fixed effects in a linear model takes the form
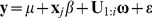
(2)

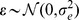
where 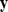 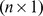 is a vector of phenotype values, 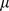 is the scalar mean term, 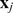 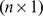 is the 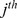 marker with scalar regression coefficient 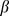, 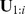 are the first 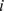 principal components with coefficient vector 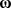 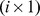, and 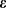 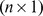 is the normally distributed residual error term with variance 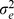. Principal components are treated as fixed effects, such that maximizing the likelihood involves directly estimating all parameters. From the Bayesian perspective of maximum *a posteriori* (MAP) estimation of all parameters, the model does not have an explicit prior on regression coefficients, 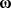, and thus implies a uniform improper prior. Furthermore, scaling each principal component by any value yields a statistically equivalent model with respect to the genetic term, 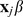, since the prior on the coefficients, 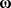, is implicitly uniform. While methods have been proposed to determine the number of relevant principal components [2], [19], in practice 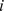 is often selected heuristically based on the eigen-spectrum or the quantile-quantile plot of the p-values from the corrected test of association.

Now consider the linear mixed model (LMM)
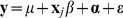
(3)

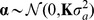


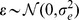
where 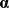 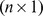 is a random effect vector with a multivariate Gaussian prior, 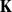 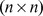 is the genetic similarity matrix between all pairs of individuals so that 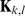 represents the similarity between individuals 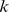 and 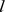, and 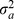 is the additive genetic variance. Here population structure is treated as a random effect and fitting the model involves integrating over the vector 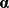 with respect to the Gaussian prior so that the likelihood is maximized with respect to 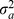, 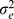, 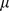, and 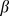 [20], [5].

For simplicity, let the genetic similarity matrix 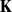 be a simple function of observed genotypes as in Patterson, et al. [2], and consider the singular value decomposition from equation (1) and the factorization of 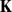


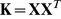
(4)

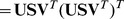


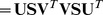


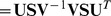


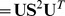


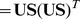


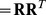
so that the columns of 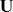 are the principal components of the genotype matrix, 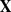, exactly as in equation (1), and, by construction, the columns of 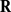 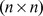 are the principal components weighted by their respective singular values. We note that each principal component 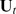 has a singular value 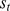 and eigen-value 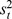. Using the property of a multivariate Gaussian that 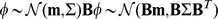, and the decompositions in (4), it is apparent that 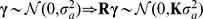, so the LMM (3) can be rewritten equivalently as
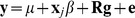
(5)

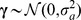


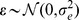
Based on the relationship between equations (2) and (5), it is apparent that modeling principal components as fixed or random effects share the same underlying regression model. This transformation explicitly formalizes the previously described relationship between modeling principal components as fixed versus random effects [14], [16], [15], [11], [21]. While the LMM includes all principal components, only 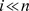 principal components are included in the fixed effects model since the number of covariates cannot be on the same order as the sample size while still maintaining reasonable statistical power in a fixed effects model [22]. We discuss the implications of this result in subsequent sections.

We note that while equation (4) assumes 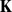 is the product of the centered and scaled genotype matrix [2], this relationship is also consistent with other genetic similarity metrics that yield a positive semi-definite 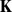. Other closely related metrics use the estimated rather than observed allele frequencies [3], adjust the similarity of an individual to itself to reduce sampling variation [23], use a Gower's centering to reduce sampling variance [4] or are proportional to these metrics [4]. In addition, each marker may be scaled or centered [9], [24], or other more complicated metrics may be used [5], [25], [26]. Finally, the simiarity metric can be constructed using only the top set of markers identified by a test of association that does not correct for population structure or kinship [6], [11], [12]. Any of these similarity metrics can be used in the LMM or the principal components of the corresponding similarity matrix can be included as fixed effects.

### Linear mixed model considers principal components' eigen-values

It is well established that the eigen-value of each principal component serves as a metric of biological relevance in relation to any underlying population structure [2], [27]. Thus a method for determining the relevance of a principal component to a given phenotype should consider both its eigen-value and its correlation with the phenotype [19]. Therefore, instead of considering only the principal components, 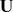, a more sophisticated model should consider the weighted principal components, 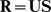, since 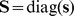 weights each principal component by its corresponding singular value (i.e. the square root of its eigen-value). However, in the fixed effect model the estimate of the genetic effect, 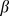, is invariant to the scale of the principal components due to the uniform prior implied in equation (2). Thus the fixed effect model assumes that each principal component has equal prior probability of being relevant to the phenotype. Alternatively, the LMM explicitly models the scale of the weighted principal components in equation (5). The LMM considers both the eigen-value and correlation with the phenotype when determining the relevance of each principal component to the phenotype. Moreover, the LMM's Gaussian prior on regression coefficients implies the biologically desirable property that a principal component with a larger eigen-value has a higher prior probability of being relevant to the phenotype [28].

### Inference methods

Since modeling principal components as fixed or random effects share the underlying regression model, the differences in their ability to account for population structure and kinship [18], [17], [1], [4] can be attributed to the different inference methods and the number of principal components included. Yet the substantial theoretical and practical consequences of these differences have not been examined. With the goal of elucidating the statistical differences between modeling principal components as fixed versus random effects, we consider the theoretical properties of exact inference methods for the LMM where 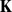 is fixed beforehand [5], [7], [9], [6]. We note that our discussion also applies to approximate LMM methods since they approximate other aspects of the model [29], [4], [8].

In both fixed and random effects models, the parameter of interest for the hypothesis test is the coefficient 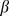 corresponding to the effect of a single genetic marker, 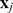, so that the coefficients 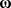 or 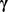 corresponding to the principal components are so-called nuisance parameters not of direct interest. The difference between the methods lies in how the statistical inference treats these nuisance parameters. The fixed effect model necessarily incorporates only 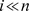 principal components and maximizes the likelihood with respect to all coefficients so that the hypothesis test is conducted at the maximum likelihood estimates of the nuisance parameters. Thus the fixed effects model implies the likelihood
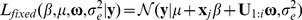
(6)which has 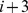 free parameters to be estimated from the data. Therefore 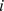 degrees of freedom are used to correct for population structure.

Alternatively, the LMM includes all principal components in the model and integrates over the random effect with respect to its prior distribution. The likelihood can be stated in terms of the genetic similarity matrix,
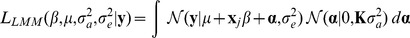
(7)[20] or equivalently in terms of the scaled principal components,
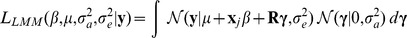
(8)based on the equivalence between equations (3) and (5). While other equivalent forms of the likelihood are used for estimation in practice [6], [5], stating the likelihood in this way formalizes the Bayesian interpretation of the LMM where a Gaussian prior is placed on the regression coefficients of the principal components and the effect of population structure and kinship is integrated out. Due to the integration over nuisance parameters, the LMM is able to include all principal components in the statistical model, yet estimate only 4 free parameters from the data.

### Dimensionality of population structure versus kinship

Population structure and kinship are both confounding factors in GWAS since they produce covariance between individuals' phenotype values. Yet the dimensionality of these two processes are different. Population structure is a low dimensional process embedded in a high dimensional space so that a relatively small number of principal components represent the underlying population genetics [2], [27], [30]. Therefore, a small number of principal components can be adequate to account for population structure in GWAS datasets [3], [1]. Conversely, kinship is a high dimensional process since small sets of individuals are very closely related while being unrelated to the remaining individuals. Consider an idealized example of independent parent-offspring duos so that the coefficient of coancestry between parent and offspring is 0.5, and 0 between all other individuals. It follows directly that the corresponding coancestry matrix is block diagonal and the eigen-spectrum has a long tail so that all eigen-values are nonzero (Figure S1). Thus kinship is a high-dimensional process that cannot be captured by a small number of principal components. Moreover, GWAS datasets contain a mixture of population structure and kinship that can produce eigen-spectra with long tails yet have very large leading eigen-values. This interpretation of kinship is consistent with the long history of modeling the full eigen-spectrum with a random effects model for trait prediction in plant and animal breeding [31], [32], [33], heritability estimation in medical genetics [33] and linkage analysis with arbitrary pedigrees [34].

### Assessing the complexity of a regression model

A generalized metric of model complexity facilitates evaluation of the theoretical and empirical properties of competing regression models. In the simple case of comparing two fixed effects models, the most natural metric is the number of parameters. Thus compared to a model with 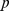 predictors, adding an additional predictor and using 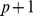 parameters produces a more complex model that will explain more of the variance in the response. Standard theory shows that increasing the number of parameters increases the covariance between the observed and fitted response [22]. In this context, the number of parameters is referred to as the degrees of freedom of the model [22].

The metric of model complexity introduced here can be generalized to arbitrary regression models with normally distributed errors. We consider the general theory first and then apply it to specific models. Letting 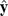 denote the fitted response, and 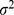 denote the variance of the random error, the “effective number of parameters” or “effective degrees of freedom” (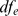) is defined as
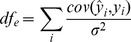
(9)and explicitly connects the model complexity with how well the model fits the response [35], [36]. It is clear that increasing the degrees of freedom causes the model to explain more of the variance in the response, thus increasing the covariance between the observed and fitted response. When the fitted response is a linear function of the observed response, such that
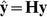
(10)where 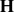 is determined by the data, then
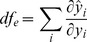
(11)

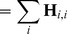
(12)

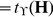
(13)
[35], [36].

Considering a fixed effects model with 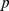 predictors in 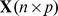, the estimated coefficients are
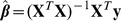
(14)so that the fitted values have the form
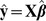
(15)

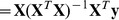
(16)

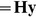
(17)where 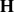 is defined by construction. The effective degrees of freedom is thus
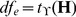
(18)

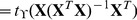
(19)

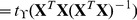
(20)

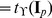
(21)

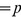
(22)so that it is equal to the number of parameters in the model, and satisfies the standard definition of degrees of freedom in the case of ordinary least squares estimation [35], [36].

This generalized theory has been widely adopted as a metric of model complexity for penalized splines, nonparametric regression and generalized additive models where 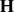 does not have such a simple form [37], [38], [39], [40], [28]. The effective degrees of freedom is thus a fundamental statistic in regression modeling that gives insight into the theoretical and empirical properties of a statistical model [38], [35], [36], [28] and much recent work has focused on developing this statistic for specific models [41], [42], [43], [44], [45], [46], [47], [48].

### Effective degrees of freedom of the linear mixed model

While the relationship between the LMM and including principal components as fixed effects has been previously discussed [14], [16], [15], [11], [21], an explicit examination of the complexity of these methods illustrates how they model the population genetics of the data. In standard GWAS analysis, population structure is modeled as a low dimensional process [2], [27], [30] and a small number of principal components are included as fixed effects [3], [1]. Following the theory from the previous section, the degrees of freedom is equal to the number of principal components included and serves as a metric of the complexity of the correction for population structure. Moreover, the degrees of freedom is fixed and determined by the analyst.

Alternatively, the LMM is able to model both population structure and kinship by considering the full eigen-spectrum. Yet assessing the model complexity is no longer trivial since all principal components are included while only 4 parameters are estimated. Therefore, we consider the effective degrees of freedom of the LMM in order to elucidate the statistical properties of the LMM as well as its biological interpretation.

Ignoring fixed effects for simplicity, the estimated the fitted response values based on only the random effect are
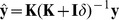
(23)

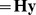
where 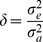 and 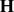 is defined by construction [20]. Following the theory from the previous section, the effective degrees of freedom is
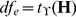
(24)

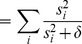
(25)where 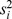 is the 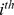 eigen-value of 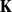 forming the diagonal of 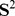 in equation (4) and the derivation of equation (25) is shown in the File S1.

This form of the effective degrees of freedom facilitates an interpretation of the influence of each principal component that is composed of a marker-based element, 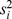, and a phenotype based element, 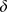. It is apparent that the Gaussian prior in the LMM causes the influence of the 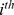 principal component to be a nonlinear function of the magnitude of its corresponding eigen-value, 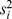. This formulation satisfies our intuition for 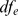 since the contribution of a single principal component is between 0 and 1 so that 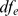, which is the sum of the contributions of all principal components, is naturally bounded between 0 and the number of principal components. Moreover, while 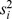 has a local effect on the influence of each principal component separately and is independent of the phenotype, estimating 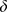 adaptively learns the effective degrees of freedom based on the correlation of the principal components with the phenotype and has a global effect by influencing the contribution of all principal components. In addition, it follows that the effective degrees of freedom of each principal component decreases with its eigen-value.

Returning to the biological interpretation of the effective degrees of freedom, we note that LMM relates the genetic similarity between individuals to the heritability of the trait [23], as well as population structure and kinship [49], [3], [4], [5], [1]. Thus the LMM uses the estimated “pseudo-heritability” of the trait in the present set of individuals to determine how strongly to correct for population structure and kinship. This data-adaptive property reflects the ability of the LMM to learn 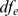 directly from the data. Moreover, the 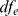 statistic is composed of heritability, population structure and kinship so that the value of 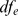 reflects the “effective dimensionality” of the correction for confounding.

### Low rank linear mixed model

To this point we have considered the standard LMM where the genetic similarity matrix is full rank and all principal components make a contribution to the phenotype [5], [4], [9], [7], [29], [8]. Yet the correction for genetic confounding due to population structure and kinship need not necessarily be full rank. Including principal components that are not biologically relevant to the given phenotype can dilute the influence of relevant principal components and degrade the quality of the correction since the random effect is governed by a single global parameter, 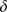. The low rank linear mixed model (LRLMM) has two distinct interpretations that depend on the nature of the genetic similarity metric. When the metric is based on markers selected using a test of association that omits population structure and kinship [6], [11], [12], the eigen-spectrum can be partitioned into principal components representing markers tagging genetic variants responsible for genetic confounding and principal components unrelated to genetic confounding. We note that such metrics consider the population genetics of the only the selected markers, rather than the entire genome. In this case, a LRLMM can learn the partition and use only a subset of principal components to correct for genetic confounding [6], [11], [12]. Alternatively, when the genetic similarity metric is based on a genome-wide set of markers and not based on the phenotype, the eigen-spectrum can be partitioned, at least in theory, into principal components representing population structure, kinship, and random noise. Here, we consider learning the partition of the principal components in this latter context using a data-adaptive LRLMM.

Learning the partition of principal components using an LRLMM requires a metric of model complexity that facilitates the comparison of models with different number of principal components. The effective degrees of freedom is a natural metric of complexity that extends to low rank models and has the form
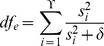
(26)where 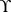 denotes the rank. Furthermore, learning the optimal rank requires a metric of how well the model fits the data and we consider Akaike Information Criterion (AIC) [50], Bayesian Information Criterion (BIC) [51], and generalized cross-validation (GCV) [52], which all depend on the effective degrees of freedom and are widely used in this context in many areas of statistics [38], [28], [53]. Moreover, we also consider the log-likelihood.

In order to identify relevant principal components, we fit a LRLMM where the rank varies from 0, where we fit the standard linear model, to the sample size, where the full rank LMM is used. Principal components are added to the model sequentially and the log-likelihood and effective degrees of freedom are evaluated for each rank. Since the order in which principal components are added to the model affects the optimal rank, we consider different orderings of the principal components based on 1) eigen-value, 2) squared correlation between principal component and phenotype (corSq), 3) eigen-value multiplied by squared correlation between principal component and phenotype (corSq*eigen-value) [19], 4) degrees of freedom from fitting each principal component individually (DF). The DF ordering involves fitting the LRLMM with rank 1 for each principal component and evaluating 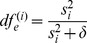 for each model. The principal components are then sorted based on their relevance to the phenotype as measured by 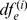.

These methods are available in the software package *genard* at http://mezeylab.cb.bscb.cornell.edu/Software.aspx.

## Results

### Simulations

We considered two distinct strategies for simulating genetic confounding and examined each separately. First, we simulated genetic confounding based directly on the principal components of a full rank genetic similarity matrix where we used 1000 European individuals from the Multi-Ethnic Study of Atherosclerosis (MESA) [54] and used Balding-Nichols metric from EMMAX [4] based on 45,000 markers pruned from 650,000. We conducted simulations to evaluate criteria for selecting the optimal rank of a LRLMM. Using the mean squared error of estimated heritability as a metric for determining how well a method modeled the data, we simulated genetic confounding based on 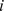 principal components of 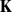 randomly sampled from the first 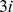 for 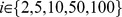. The phenotype was simulated by sampling coefficient values for each principal component from 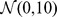 for heritabilities of 30, 40, 50 and 60%, and we considered 1000 replicates for each condition. We evaluated the mean squared error from sorting principal components by eigen-value, corSq, corSq*eigen-value and DF, and using AIC, BIC, GCV, or −2*log-likelihood to select the optimal rank (Figure S2, S3, S4, S5, S6, S7). These simulations indicate that selecting the rank with BIC and sorting eigen-values based on corSq, corSq*eigen-value or DF provided the most accurate estimates of heritability and thus provide the best fit to the simulated data. We used BIC for all subsequent applications of our LRLMM methods.

**Figure 1:**
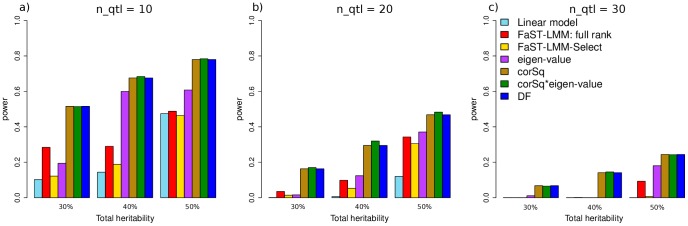
Simulation results showing power where genetic confounding is simulated directly from principal components. Power is shown at a false discovery rate of 5% for 50 replicate simulations based on 650,000 markers from 1000 European individuals from the Multi-Ethnic Study of Atherosclerosis (MESA) for total heritabilities of 30, 40 or 50%, and **a**) 10, **b**) 20 or **c**) 30 markers contributing to the phenotype. Results are shown for a linear model, FaST-LMM with a full rank genetic similarity matrix [6], FaST-LMM-Select [11], [12], and the low rank linear mixed model with 4 orderings of the principal components.

We evaluated the statistical power of multiple methods under this model of genetic confounding for the same genetic data. We simulated genetic confounding by randomly selecting 10 principal components from the first 30 to affect the phenotype and drawing coefficients from 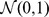 so that the principal components explained 15% of the variation in phenotype for each of 50 replicates. We considered cases where 10, 20 or 30 markers contributed to variation in the phenotype with coefficients drawn from 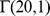 and we simulated total heritabilities of 30, 40 or 50%. A marker was considered a false positive if it had 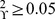 with a causal marker [55], [56], and multiple such markers in a 100 kb cluster were counted as a single false positive. True positive markers were determined by complementary criteria with the same cutoff values. Since a causal marker may be tagged by multiple true positive markers, power was defined as the number of causal markers tagged by true positives. Under these conditions, our LRLMM methods, specifically sorting principal components using corSq, corSq*eigen-value and DF orderings, were more powerful than ordering by eigen-value or other methods including a linear model with no principal components, a full rank LMM implemented in FaST-LMM [6] or a low rank LMM termed FaST-LMM-Select that constucts principal components from the top scoring markers from a linear model [11], [12] (Figure 1). We note that the increased power of our LRLMM methods that use principal components from a genome-wide set of markers is consistent with the fact that genetic confounding was based on the principal components in these simulations.

**Figure 2:**
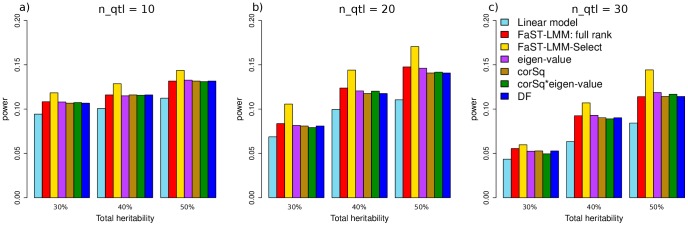
Simulation results showing power where genetic confounding is simulated based on stratified markers. Power is shown at a false discovery rate of 5% for 50 replicate simulations based on 650,000 markers from 1000 European individuals from the Multi-Ethnic Study of Atherosclerosis (MESA) for total heritabilities of 30, 40 or 50%, and **a**) 10, **b**) 20 or **c**) 30 markers contributing to the phenotype. Results are shown for a linear model, FaST-LMM with a full rank genetic similarity matrix [6], FaST-LMM-Select [11], [12], and the low rank linear mixed model with 4 orderings of the principal components.

We considered a second model of genetic confounding due to the effects of stratified markers. We evaluated the statistical power on the same genetic data where we simulated phenotypes by selecting the 5 markers most correlated with each of the first 10 principal components. Coefficients corresponding to these 50 stratified markers were drawn from 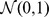. We considered cases with an additional 10, 20 or 30 randomly select markers contributing to the phenotype with coefficients were drawn from 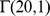 and we simulated total heritabilities of 30, 40 or 50%. Under this model of genetic confounding, FaST-LMM-Select [11], [12] performed best under all conditions (Figure 2). We note that the increased power of FaST-LMM-Select is consistent with the fact that genetic confounding was based on a small set of stratified markers, as is assumed by this method.

**Figure 3:**
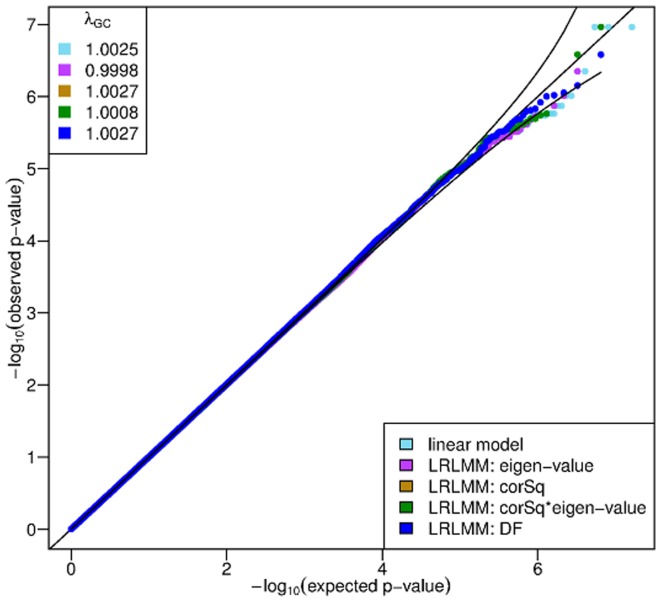
Null simulations show that our LRLMM methods do not deviate from the nominal false positive rate.

In order to examine the type I error, we sampled phenotypes from 1000 individuals from 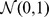 and evaluated 10 replicate simulations with our LRLMM methods using the genetic similarity matrix from the previous simulations. The quantile-quantile plots and genomic control values [13] show no deviation from the nominal false positive rate (Figure 3).

### Data analysis

**Figure 4:**
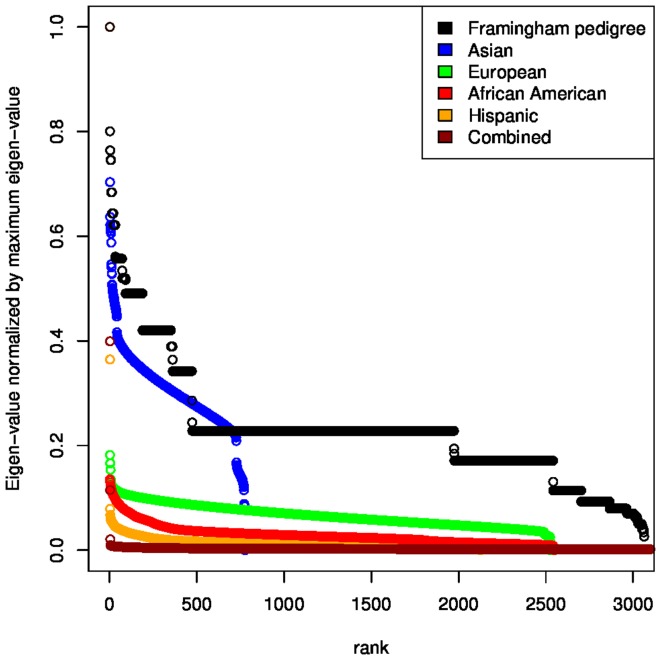
Comparison of eigen-spectra due to population structure and kinship. The eigen-spectrum based on the known pedigree from 3063 individuals from the Framingham Heart Study reflects kinship, while the eigen-spectrum for four populations from the Multi-Ethnic Study of Atherosclerosis (MESA) reflects both population structure and kinship. Eigen-values for each dataset are normalized by the maximum eigen-value so that each spectrum has a maximum of 1.

**Figure 5:**
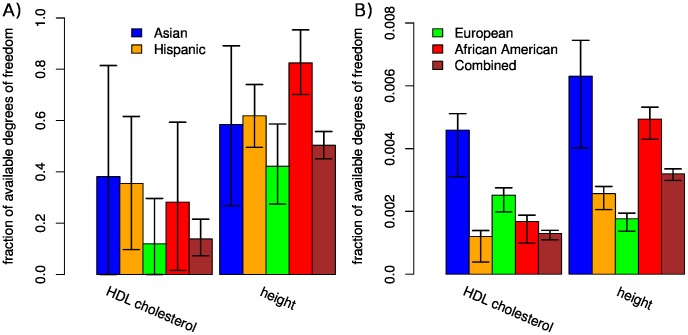
Fraction of available degrees of freedom used to account for population structure and kinship. Results are shown for A) the linear mixed model (LMM) and B) the low rank linear mixed model (LRLMM) sorting by degrees of freedom of each principal component fit individually (LRLMM-DF). Effective degrees of freedom normalized by sample size are shown for two phenotypes and four populations from the Multi-Ethnic Study of Atherosclerosis (MESA) plus the combined dataset. Error bars indicate 95% confidence intervals based on the log-likelihood surface of 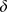. Note the large difference in the scales between (A) and (B). These approximate confidence intervals were generated by maximizing the log-likelihood of the linear mixed model (8) with respect to all parameters, determining 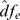 according to 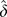 and the mapping (26), evaluating 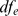 on a fine grid of values by changing the value of 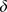, and identifying an asymmetric interval around 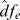 so that a standard asymptotic likelihood ratio test using a 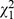 null distribution produced a 5% type I error. These confidence intervals may not be statistically optimal, but we show them here for illustrative rather than quantitative purposes.

Our analysis of GWAS data from four populations and two phenotypes from the Multi-Ethnic Study of Atherosclerosis (MESA) [54] (Table 1) illustrates properties of the LMM and demonstrates the ability of the LRLMM to boost the strength of an association signal. Eigen-spectra of the genetic similarity matrices from four MESA populations as well as the matrix of coancestry coefficients based on the known pedigree from the Framingham Heart Study [57] illustrate the different dimensionality of population structure and kinship (Figure 4). It is apparent that population structure is low dimensional so the eigen-values decay very quickly in the MESA populations, while kinship from the Framingham pedigree shows a very long tail indicative of a high-dimensional process. This observation is consistent with the results of our previous idealized example (Figure S1). In addition, the LMM relates the eigen-spectrum of the genetic similarity matrix to the phenotype and its heritability, and this relationship is reflected by the effective degrees of freedom for each principal component (Figure S8). Thus the effective degrees of freedom, normalized by the sample size, used by the LMM for height is substantially larger than for HDL cholesterol (Figure 5A), since height is known to have a larger heritability [58], [59]. Moreover, the fact that the effective degrees of freedom is a substantial fraction of the sample size indicates that the LMM models the high-dimensional confounding effect of kinship. We note that the heterogeneity among populations can be attributed either to differential population structure or kinship, or to stochastic effects. Finally, we note that the LMM was fit by maximum likelihood here, but estimation by REML has little effect (Figure S9).

**Table 1:** Sample size for each population and phenotype from the Multi-Ethnic Study of Atherosclerosis (MESA) dataset.

	Asian	Hispanic	European	African American	combined
HDL cholesterol	772	1436	2481	1584	6273
height	775	2104	2522	2528	7929

**Figure 6:**
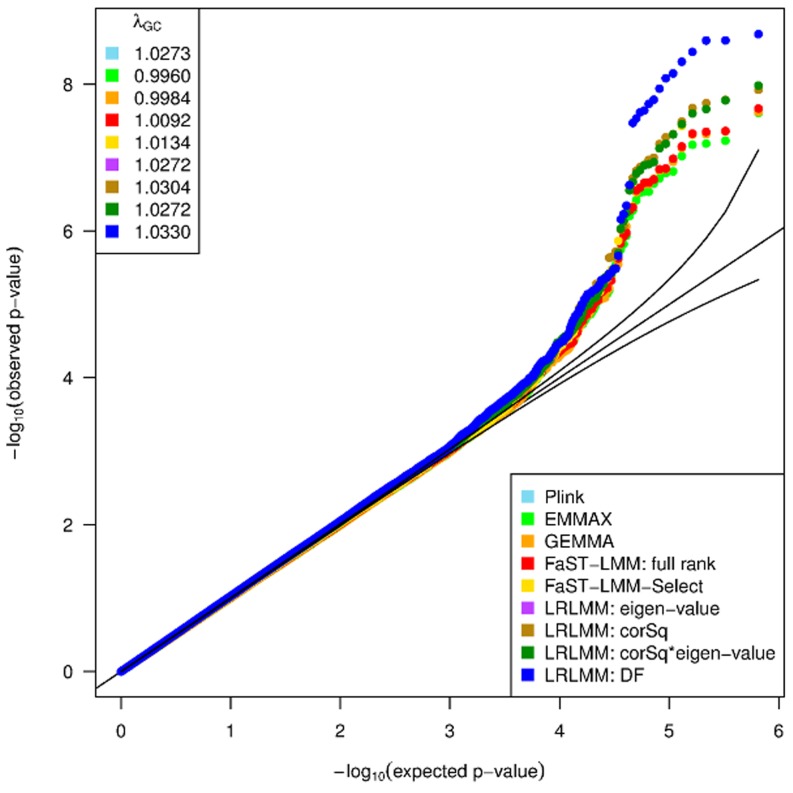
Quantile-quantile plot for association tests for HDL cholesterol in Europeans. Results are shown from a standard linear model (Plink [60]), 4 versions of the linear mixed model (EMMAX [4], GEMMA [9], FaST-LMM [6]), FaST-LMM-Select [11], [12]), and the low rank linear mixed model with 4 orderings of the principal components. We note that LRLMM using eigen-value and corSq*eigen-value orderings selected no principal components correction and thus give the same p-values as Plink. 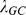 indicates the genomic control value [13].

**Figure 7:**
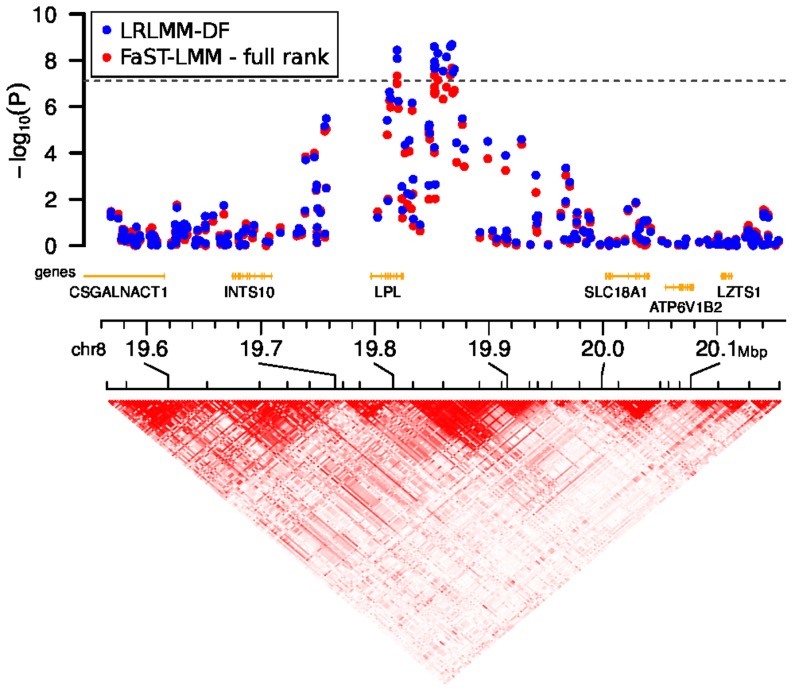
Manhattan plot of chromosome 8 showing 19.6 Mbp to 20.1 Mbp. Results are shown for the low-rank linear mixed model (LRLMM) ordering principal components by degrees of freedom based on the fit of LRLMM with each principal component individually (LRLMM-DF). P-values from FaST-LMM [6] are shown for comparison. Dashed line indicates Bonferroni correction of 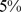 for 650,000 markers. Linkage disequilibrium is shown in terms of D′. We note that other methods are omitted for the sake of clarity.

Applying the LRLMM sorting by degrees of freedom from fitting each principal component individually (LRLMM-DF) selects effective degrees of freedom that are substantially smaller than for the full rank model and the effective degrees of freedom is generally larger for height than for HDL cholesterol (Figure 5B). Moreover, the width of the 95% confidence interval is also substantially smaller. Applying the LRLMM-DF for association testing for HDL cholesterol in Europeans substantially boosts the signal from markers on chr8 between positions 19,852,309 and 19,869,675 compared to a standard linear model (Plink [60]) and three versions of the full rank LMM (EMMAX [4], GEMMA [9], FaST-LMM [6]), and FaST-LMM-Select [11], [12] (Figure 6, S10). The boost in the association signal is apparent in a zoom-in manhattan plot illustrating that the LRLMM-DF method produces many more p-values that exceed the Bonferroni cutoff (Figure 7). This region has previously been associated with HDL cholesterol [61], [62], so LRLMM-DF is able to strengthen the signal of a replicated association. Analysis of 2481 European individuals for 650,000 markers took 12 minutes and 6273 combined samples took 2 hours 43 minutes on an 8 core Intel®Xeon®E5450.

## Discussion

The linear mixed model (LMM) has become a standard method to account for the confounding effects of population structure and kinship in GWAS datasets [4], [1], [6], [7], [8], [9], [10], [11], [12]. Our theoretical and empirical analysis illustrates the properties of the LMM and formalizes a biological interpretation of the model. We introduced the effective degrees of freedom in order to interpret model complexity of the low rank LMM (LRLMM) and the strength of the correction for population structure and kinship. A fixed effects model can include relatively few principal components, yet the LMM models the entire eigen-spectrum of the genetic similarity matrix. Thus while it is generally suggested that the degrees of freedom in a regression model be on the order of the square-root of the sample size in order to maintain reasonable statistical power [22], the effective degrees of freedom of the full rank LMM routinely exceeded 40% of the sample size in our analysis and reached up to 80%. The effect of using such high effective degrees of freedom on the statistical test of association remains an open question. Moreover, wide confidence intervals for the effective degrees of freedom indicate that there is a high degree of uncertainly about the strength of the correction for population structure and kinship. In contrast, the confidence intervals for the LRLMM are substantially smaller and are thus less influenced by stochastic effects. These results indicate that a high-dimensional correction for confounding may benefit from a fully Bayesian treatment of the linear mixed model as it would integrate over the uncertainty of the strength of the correction [24]. Yet the LRLMM would likely not benefit as much since it produces a low-dimensional fit to the data.

The ability of our low rank linear mixed model (LRLMM) to boost the signal of a known association for HDL cholesterol in Europeans indicates that the LMM can overfit the data so that the random effect absorbs too much of the phenotype variance. If the true model is low rank, then the LRLMM will have greater power than the LMM. Alternatively, if the true model is high-dimensional then the full rank LMM is more appropriate. Since there is no way to know true dimensionality *a priori*, our novel LRLMM provides an alternative test of association that can boost the strength of an association or identify additional associations if it is a better fit to the data.

The LMM has been interpreted in the context of kinship [31], [23], [63], genetic background [64], [11], [63], latent environmental effects [49], [12], highly differentiated markers in the context of population structure [21], [65], [1], and correcting for confounding in the context of rare variants [12]. Moreover, the formulation of the LMM in terms of genetic background underlies the motivation of a LRLMM based on markers selected from a preliminary test of association [11], [12]. Here, we have formalized the interpretation of genetic confounding and the LMM in terms of the population genetics of both population structure and kinship. The LRLMM developed here is based on the principal components from a genome-wide set of markers and the low rank corresponds to the relevance of only a subset of principal components. We note that our simulations show that a given method works best when the genetic architecture follows the assumptions underlying the method's statistical model.

With the growing interest in testing associations of rare variants, new problems of genetic confounding are arising due to the more recent origin and more localized distribution of rare compared to common variants [66], [67], [68]. While addressing this challenge will require extensive methodology development and empirical investigations, the framework discussed here suggests important issues to consider in order to apply appropriate corrections for genetic confounding in the next-generation of GWAS.

## Supporting Information

Figure S1 **Simulated genetic similarity matrices and their eigen-spectra.** **a**) The eigen-spectrum of 3 distinct populations is dominated by the the first 3 eigen-values. **b**) Kinship represented by 33 parent-offspring duos has a long tailed eigen-spectrum. **c**) The weighted sum of the genetic similarity matrices from (a) and (b) combine population structure and kinship so that the eigen-spectrum has a long tail, yet is dominated by the first 3 eigen-values. We note that the eigen-spectra are scaled by the largest eigen-value so that all spectra have the same scale. Moreover, we note that for simplicity the genetic similarity matrices were constructed directly and are not based on real or simulated genotype data.
(TIFF)

Figure S2
**Estimated heritability based on 6 LMM methods for** 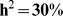 **.** Estimated heritability is shown for 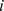 relevant principal components sampled randomly from the first 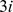 principal components for 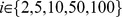. Results are shown for the low rank linear mixed model (LRLMM) using only the relevant principal components (True), the full rank LMM (Full) and the LRLMM using 4 orderings of the principal components: eigen-value, corSq, corSq*eigen-value and DF. Results are shown where the optimal rank for the LRLMM was determined by minimizing the AIC, BIC, Generalized Cross Validation (GCV) or −2*log-likelihood (logLik). The dashed line on each plot shows the true heritability.(TIFF)

Figure S3
**Estimated heritability based on 6 LMM methods for** 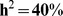 **.** Estimated heritability is shown for 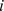 relevant principal components sampled randomly from the first 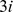 principal components for 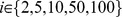. Results are shown for the low rank linear mixed model (LRLMM) using only the relevant principal components (True), the full rank LMM (Full) and the LRLMM using 4 orderings of the principal components: eigen-value, corSq, corSq*eigen-value and DF. Results are shown where the optimal rank for the LRLMM was determined by minimizing the AIC, BIC, Generalized Cross Validation (GCV) or −2*log-likelihood (logLik). The dashed line on each plot shows the true heritability.(TIFF)

Figure S4
**Estimated heritability based on 6 LMM methods for** 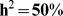 **.** Estimated heritability is shown for 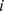 relevant principal components sampled randomly from the first 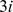 principal components for 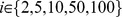. Results are shown for the low rank linear mixed model (LRLMM) using only the relevant principal components (True), the full rank LMM (Full) and the LRLMM using 4 orderings of the principal components: eigen-value, corSq, corSq*eigen-value and DF. Results are shown where the optimal rank for the LRLMM was determined by minimizing the AIC, BIC, Generalized Cross Validation (GCV) or −2*log-likelihood (logLik). The dashed line on each plot shows the true heritability.(TIFF)

Figure S5
**Estimated heritability based on 6 LMM methods for** 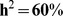 **.** Estimated heritability is shown for 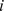 relevant principal components sampled randomly from the first 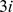 principal components for 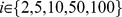. Results are shown for the low rank linear mixed model (LRLMM) using only the relevant principal components (True), the full rank LMM (Full) and the LRLMM using 4 orderings of the principal components: eigen-value, corSq, corSq*eigen-value and DF. Results are shown where the optimal rank for the LRLMM was determined by minimizing the AIC, BIC, Generalized Cross Validation (GCV) or −2*log-likelihood (logLik). The dashed line on each plot shows the true heritability.(TIFF)

Figure S6 **Mean squared error of estimated heritability across all simulation conditions for low rank linear mixed model (LRLMM).** Plots shown here summarize the results of Figures S2, S3, S4, S5 in terms of mean squared error. Results are shown for the low rank linear mixed model (LRLMM) using only the relevant principal components (True) and the LRLMM using 4 orderings of the principal components: eigen-value, corSq, corSq*eigen-value and DF. Results are shown where the optimal rank for the LRLMM was determined by minimizing AIC, BIC, Generalized Cross Validation (GCV) or −2*log-likelihood (logLik). Results from the full rank LMM are shown in Figure S14 since the mean squared errors are much larger when the true model is low rank.
(TIFF)

Figure S7 **Mean squared error of estimated heritability across all simulation conditions for full rank LMM.** Results are shown for the same simulations as in Figure S6. Results are shown for a range of heritabilities and number of relevant principal components. The results for the full rank LMM are shown here since the mean square error is substantially larger than for LRLMM methods when the true model is low rank.
(TIFF)

Figure S8 **Effective degrees of freedom for each principal component based on a linear mixed model (LMM) analysis of HDL cholesterol for four populations from the Multi-Ethnic Study of Atherosclerosis (MESA) dataset.** Total effective degrees of freedom for each population are shown in the legend.
(TIFF)

Figure S9 **Fraction of available degrees of freedom used by the linear mixed model (LMM) to account for population structure and kinship estimated using restricted maximum likelihood (REML).** Effective degrees of freedom normalized by sample size are show for six phenotypes and four populations from the Multi-Ethnic Study of Atherosclerosis (MESA) plus the combined dataset. Error bars indicate 95% confidence intervals based on the log-likelihood surface.
(TIFF)

Figure S10 **Manhattan plots for HDL cholesterol in Europeans from the Multi-Ethnic Study of Atherosclerosis (MESA).** Results shown using Plink, EMMAX, GEMMA, FaST-LMM: full rank, FaST-LMM-SELECT and our low rank linear mixed model sorting by degrees of freedom from fitting each principal component individually (LRLMM-DF).
(TIFF)

File S1 Eective degrees of freedom.
(PDF)
