# Supplementary figures and images for: Correcting for Population Structure and Kinship Using the Linear Mixed Model: Theory and Extensions
Source: PLoS One. 2013 Oct 28;8(10):e75707. doi: 10.1371/journal.pone.0075707 (PMC3810480; doi:10.1371/journal.pone.0075707)

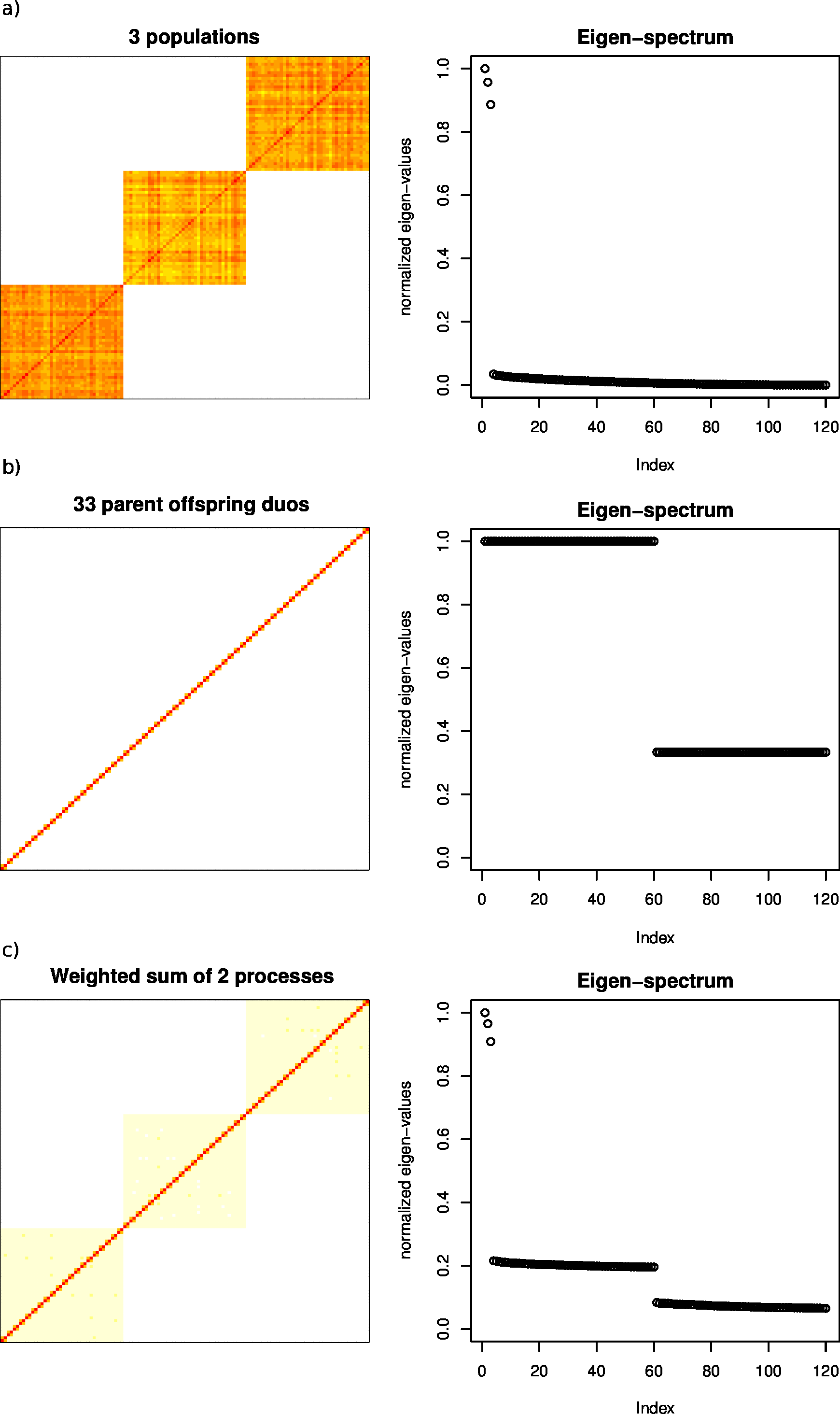

Supplement: Figure S1 — Simulated genetic similarity matrices and their eigen-spectra. a) The eigen-spectrum of 3 distinct populations is dominated by the the first 3 eigen-values. b) Kinship represented by 33 parent-offspring duos has a long tailed eigen-spectrum. c) The weighted sum of the genetic similarity matrices from (a) and (b) combine population structure and kinship so that the eigen-spectrum has a long tail, yet is dominated by the first 3 eigen-values. We note that the eigen-spectra are scaled by the largest eigen-value so that all spectra have the same scale. Moreover, we note that for simplicity the genetic similarity matrices were constructed directly and are not based on real or simulated genotype data. (TIFF) [file pone.0075707.s001.tiff]

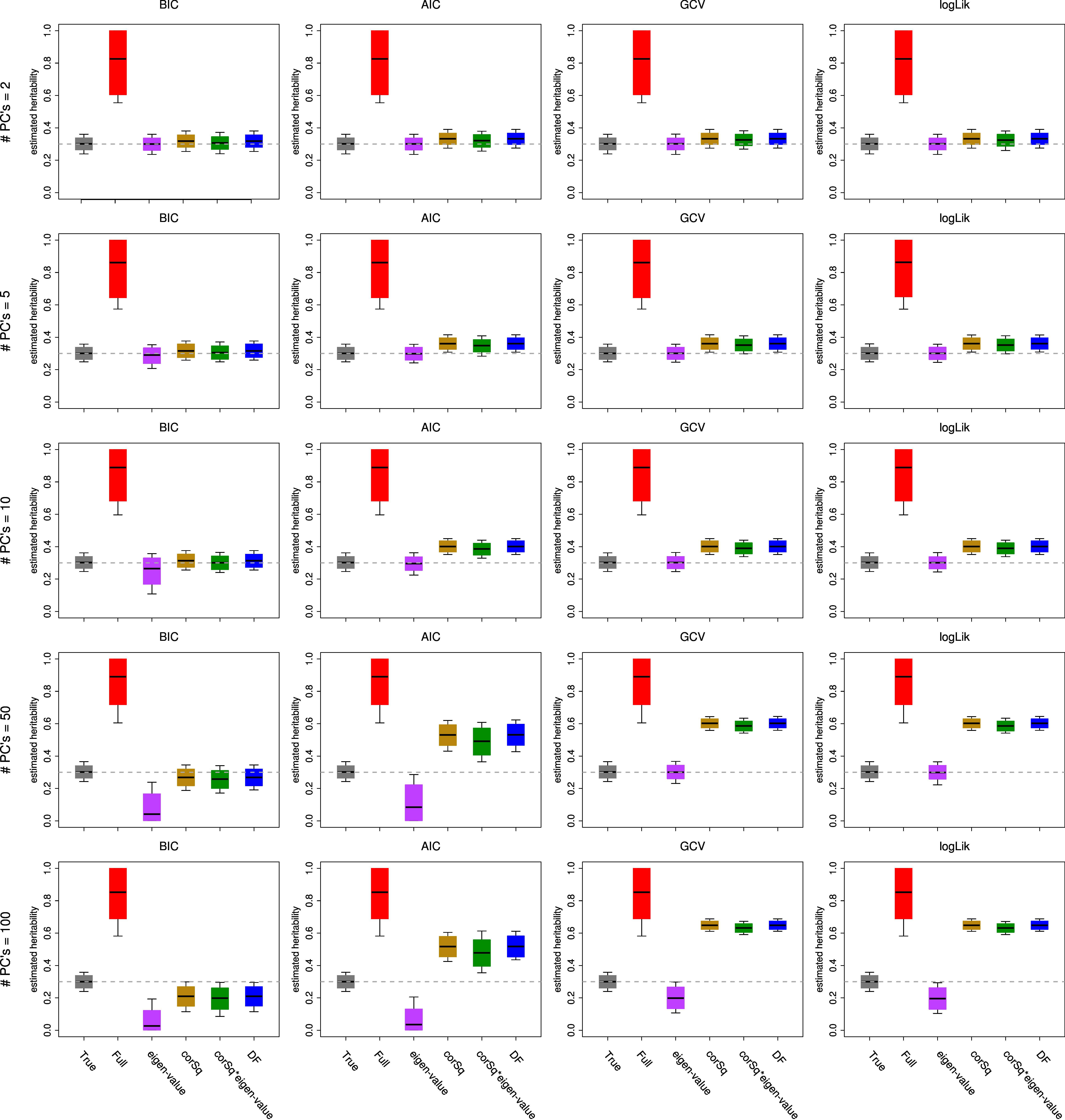

Supplement: Figure S2 — Estimated heritability based on 6 LMM methods for . Estimated heritability is shown for relevant principal components sampled randomly from the first principal components for . Results are shown for the low rank linear mixed model (LRLMM) using only the relevant principal components (True), the full rank LMM (Full) and the LRLMM using 4 orderings of the principal components: eigen-value, corSq, corSq*eigen-value and DF. Results are shown where the optimal rank for the LRLMM was determined by minimizing the AIC, BIC, Generalized Cross Validation (GCV) or −2*log-likelihood (logLik). The dashed line on each plot shows the true heritability. (TIFF) [file pone.0075707.s002.tiff]

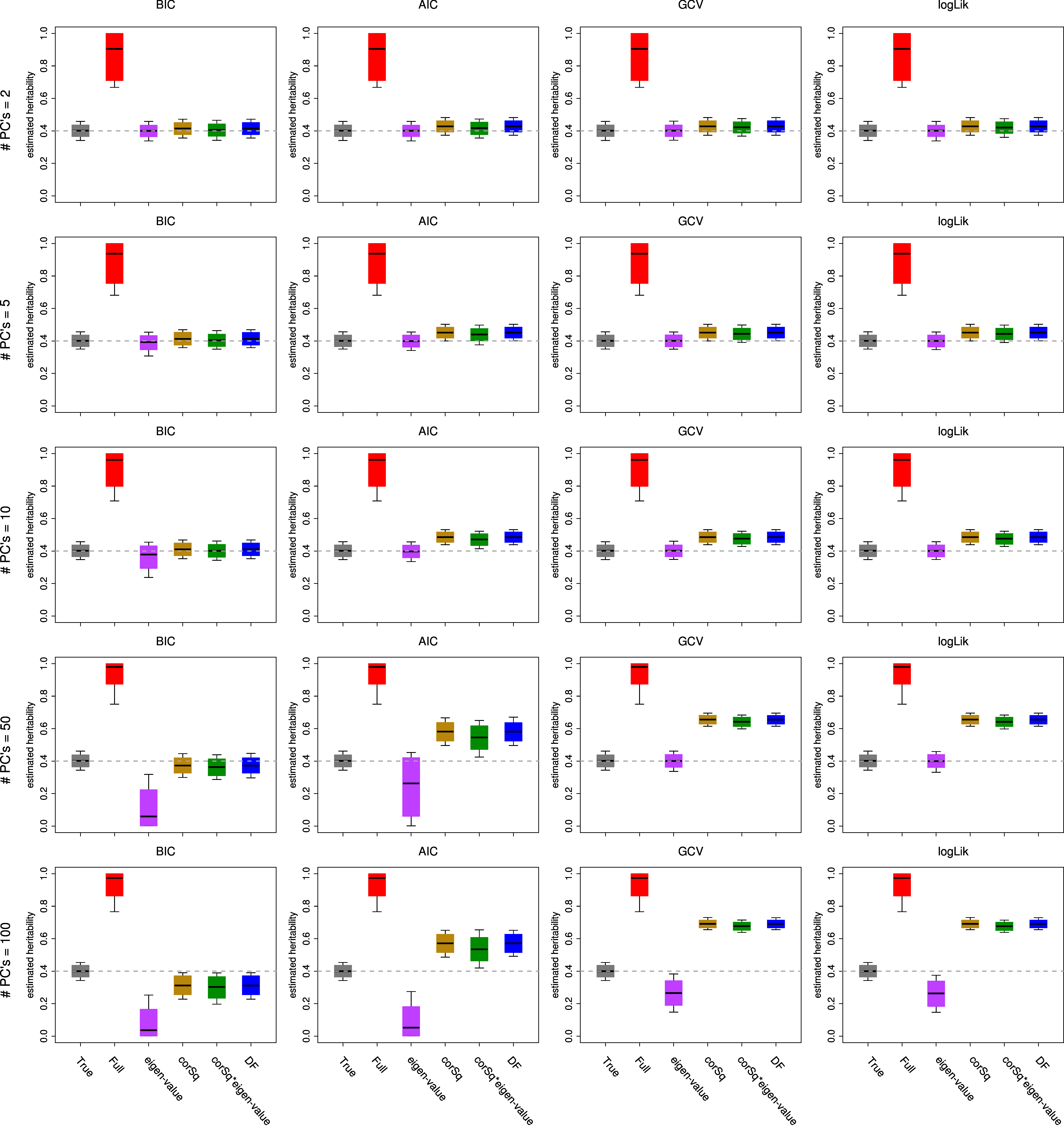

Supplement: Figure S3 — Estimated heritability based on 6 LMM methods for . Estimated heritability is shown for relevant principal components sampled randomly from the first principal components for . Results are shown for the low rank linear mixed model (LRLMM) using only the relevant principal components (True), the full rank LMM (Full) and the LRLMM using 4 orderings of the principal components: eigen-value, corSq, corSq*eigen-value and DF. Results are shown where the optimal rank for the LRLMM was determined by minimizing the AIC, BIC, Generalized Cross Validation (GCV) or −2*log-likelihood (logLik). The dashed line on each plot shows the true heritability. (TIFF) [file pone.0075707.s003.tiff]

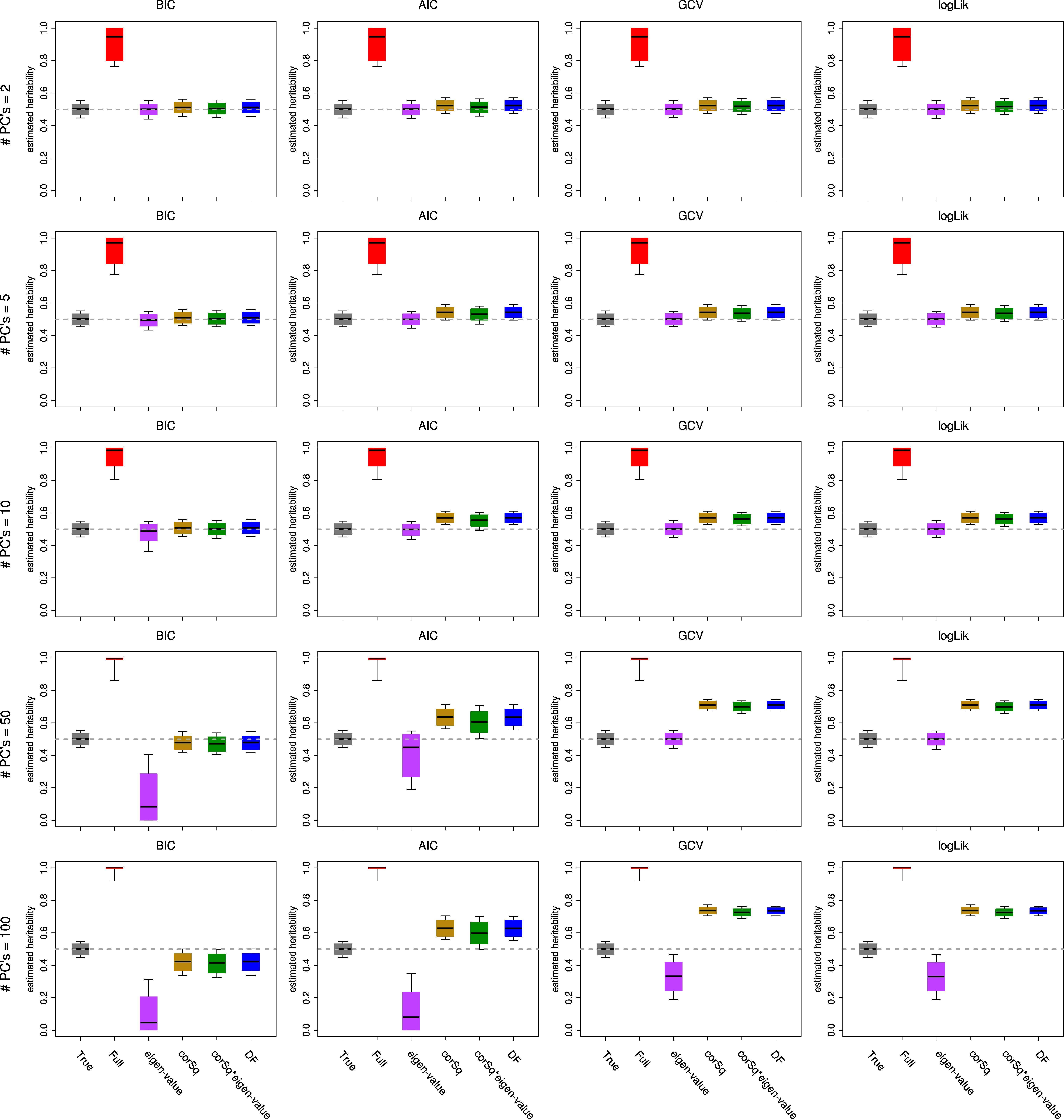

Supplement: Figure S4 — Estimated heritability based on 6 LMM methods for . Estimated heritability is shown for relevant principal components sampled randomly from the first principal components for . Results are shown for the low rank linear mixed model (LRLMM) using only the relevant principal components (True), the full rank LMM (Full) and the LRLMM using 4 orderings of the principal components: eigen-value, corSq, corSq*eigen-value and DF. Results are shown where the optimal rank for the LRLMM was determined by minimizing the AIC, BIC, Generalized Cross Validation (GCV) or −2*log-likelihood (logLik). The dashed line on each plot shows the true heritability. (TIFF) [file pone.0075707.s004.tiff]

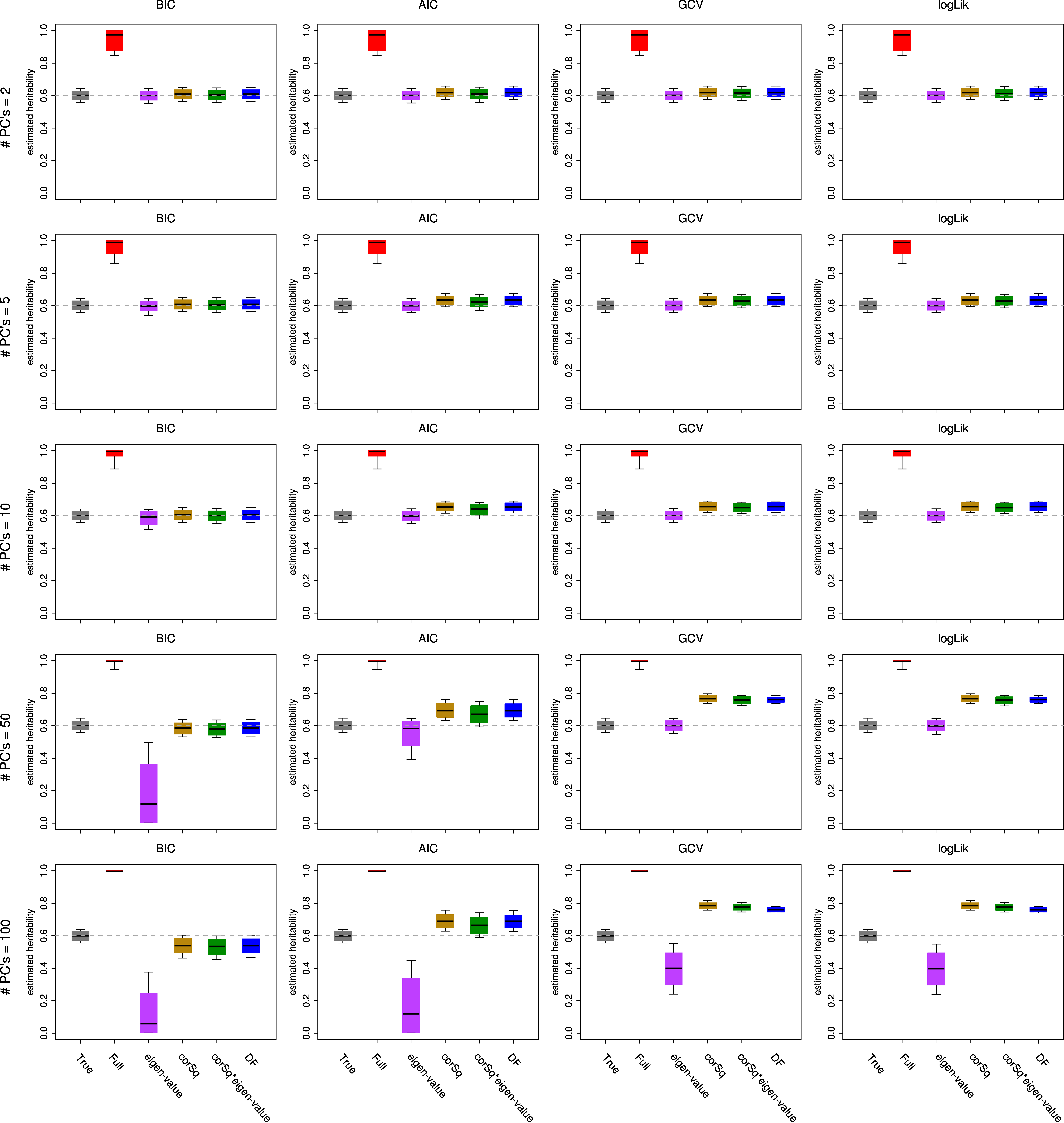

Supplement: Figure S5 — Estimated heritability based on 6 LMM methods for . Estimated heritability is shown for relevant principal components sampled randomly from the first principal components for . Results are shown for the low rank linear mixed model (LRLMM) using only the relevant principal components (True), the full rank LMM (Full) and the LRLMM using 4 orderings of the principal components: eigen-value, corSq, corSq*eigen-value and DF. Results are shown where the optimal rank for the LRLMM was determined by minimizing the AIC, BIC, Generalized Cross Validation (GCV) or −2*log-likelihood (logLik). The dashed line on each plot shows the true heritability. (TIFF) [file pone.0075707.s005.tiff]

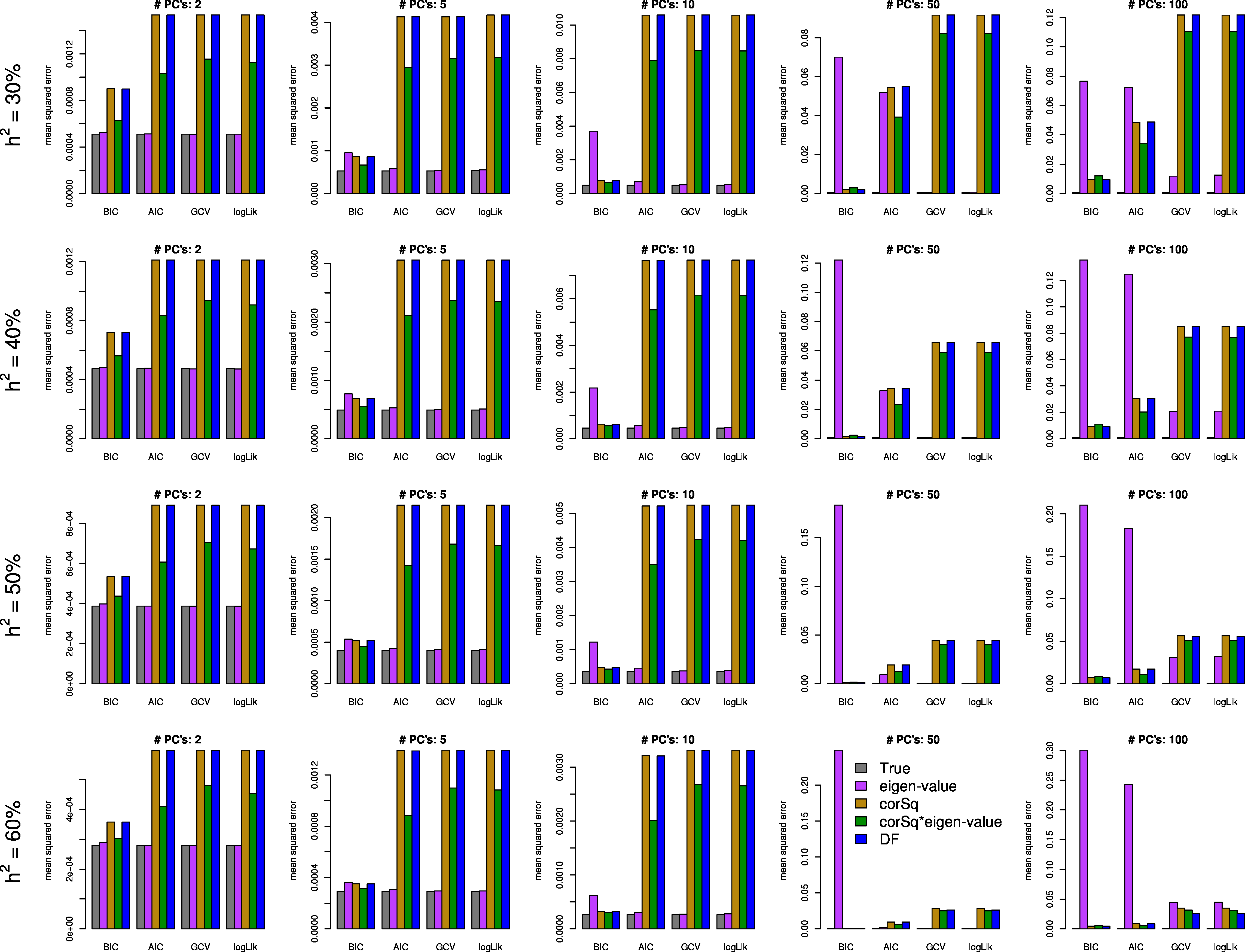

Supplement: Figure S6 — Mean squared error of estimated heritability across all simulation conditions for low rank linear mixed model (LRLMM). Plots shown here summarize the results of Figures S2, S3, S4, S5 in terms of mean squared error. Results are shown for the low rank linear mixed model (LRLMM) using only the relevant principal components (True) and the LRLMM using 4 orderings of the principal components: eigen-value, corSq, corSq*eigen-value and DF. Results are shown where the optimal rank for the LRLMM was determined by minimizing AIC, BIC, Generalized Cross Validation (GCV) or −2*log-likelihood (logLik). Results from the full rank LMM are shown in Figure S14 since the mean squared errors are much larger when the true model is low rank. (TIFF) [file pone.0075707.s006.tiff]

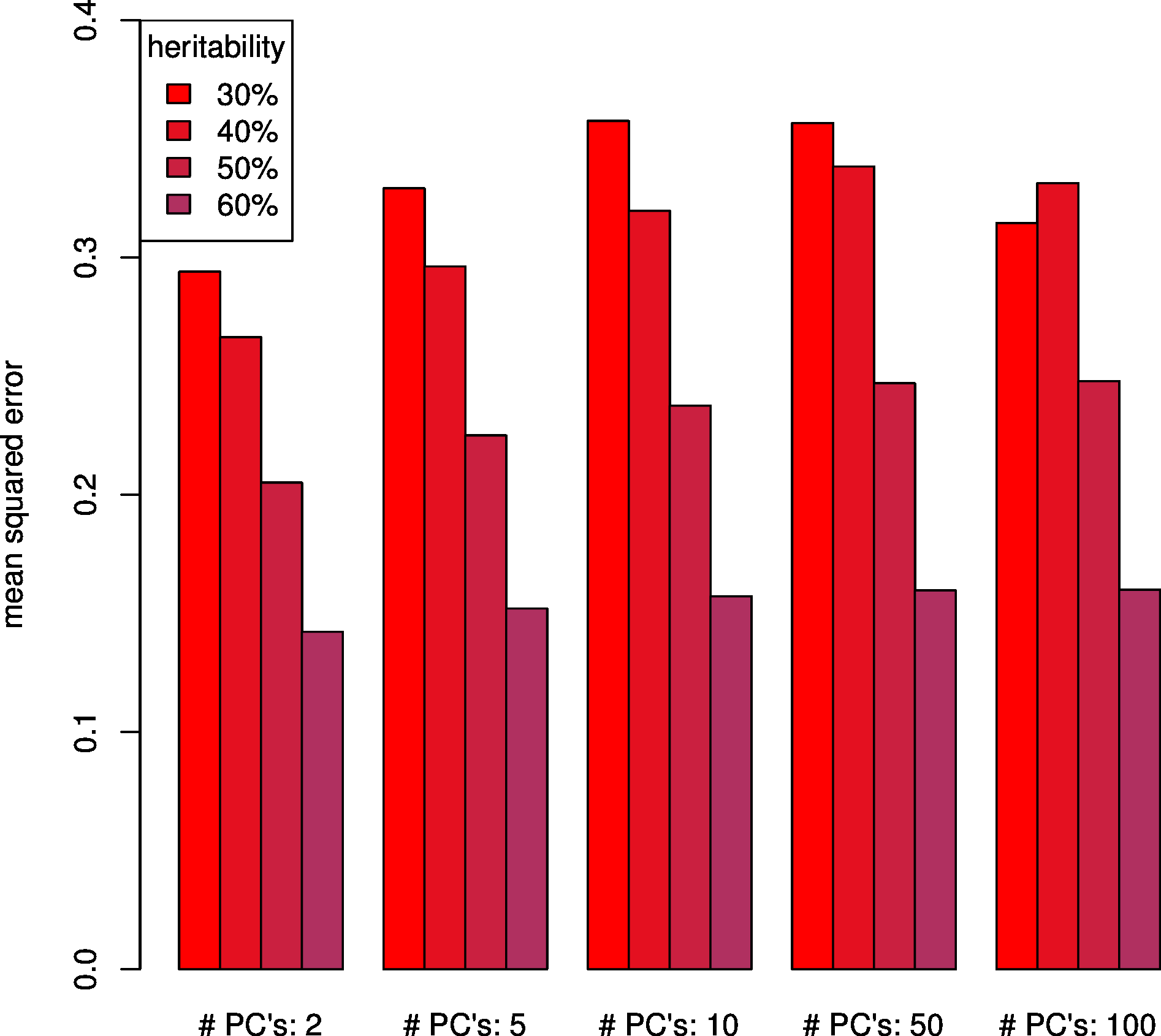

Supplement: Figure S7 — Mean squared error of estimated heritability across all simulation conditions for full rank LMM. Results are shown for the same simulations as in Figure S6. Results are shown for a range of heritabilities and number of relevant principal components. The results for the full rank LMM are shown here since the mean square error is substantially larger than for LRLMM methods when the true model is low rank. (TIFF) [file pone.0075707.s007.tiff]

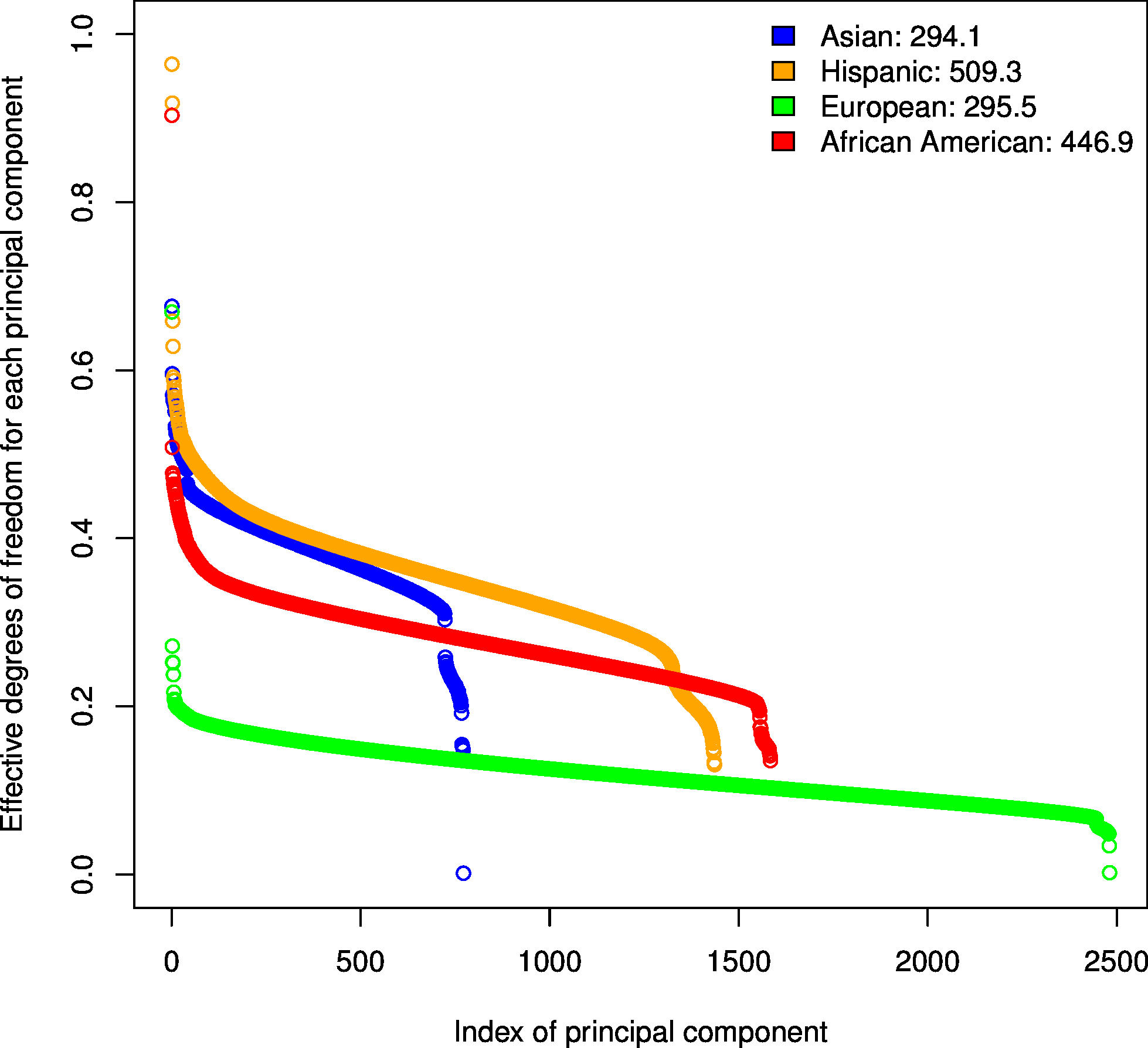

Supplement: Figure S8 — Effective degrees of freedom for each principal component based on a linear mixed model (LMM) analysis of HDL cholesterol for four populations from the Multi-Ethnic Study of Atherosclerosis (MESA) dataset. Total effective degrees of freedom for each population are shown in the legend. (TIFF) [file pone.0075707.s008.tiff]

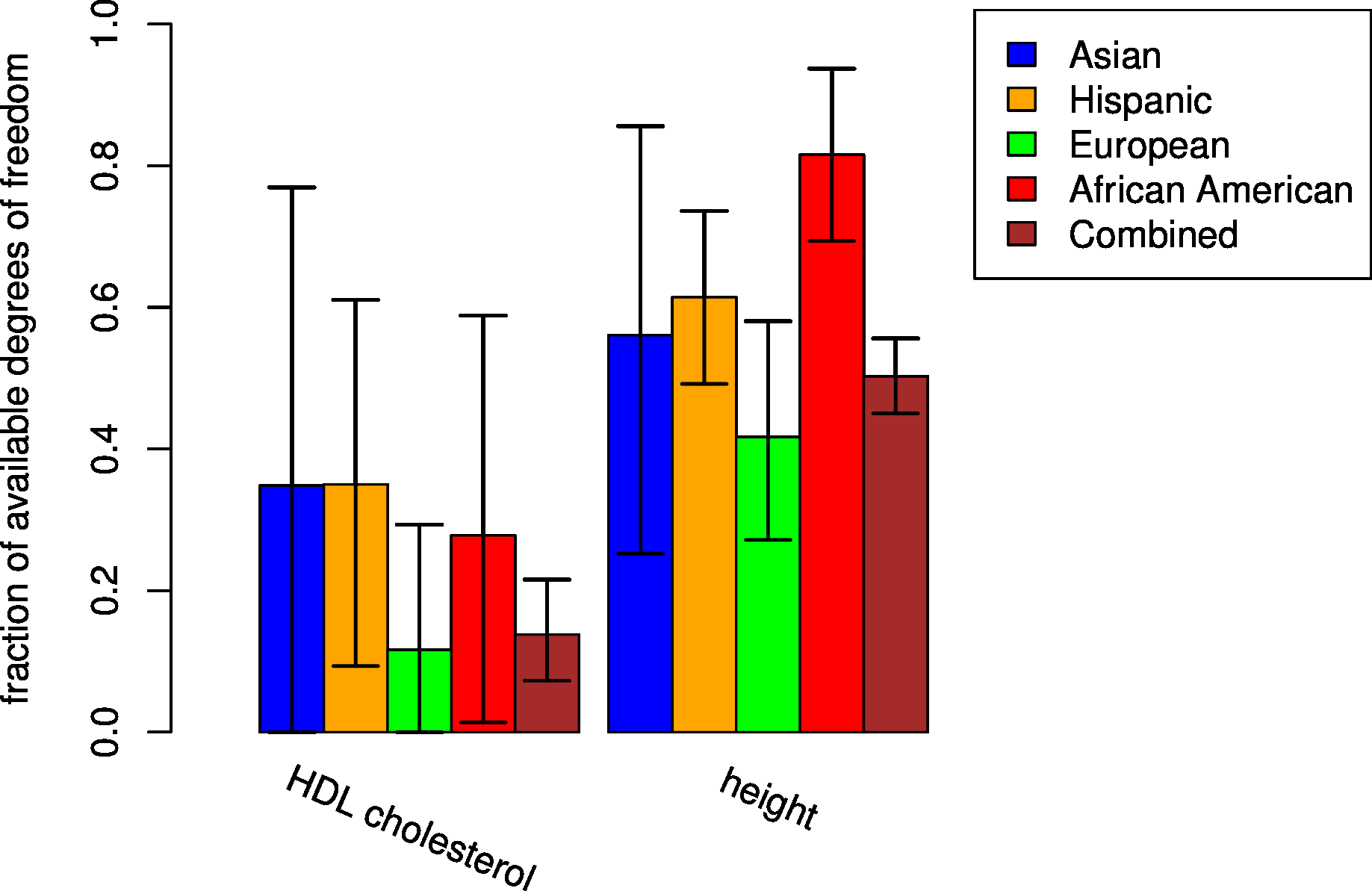

Supplement: Figure S9 — Fraction of available degrees of freedom used by the linear mixed model (LMM) to account for population structure and kinship estimated using restricted maximum likelihood (REML). Effective degrees of freedom normalized by sample size are show for six phenotypes and four populations from the Multi-Ethnic Study of Atherosclerosis (MESA) plus the combined dataset. Error bars indicate 95% confidence intervals based on the log-likelihood surface. (TIFF) [file pone.0075707.s009.tiff]

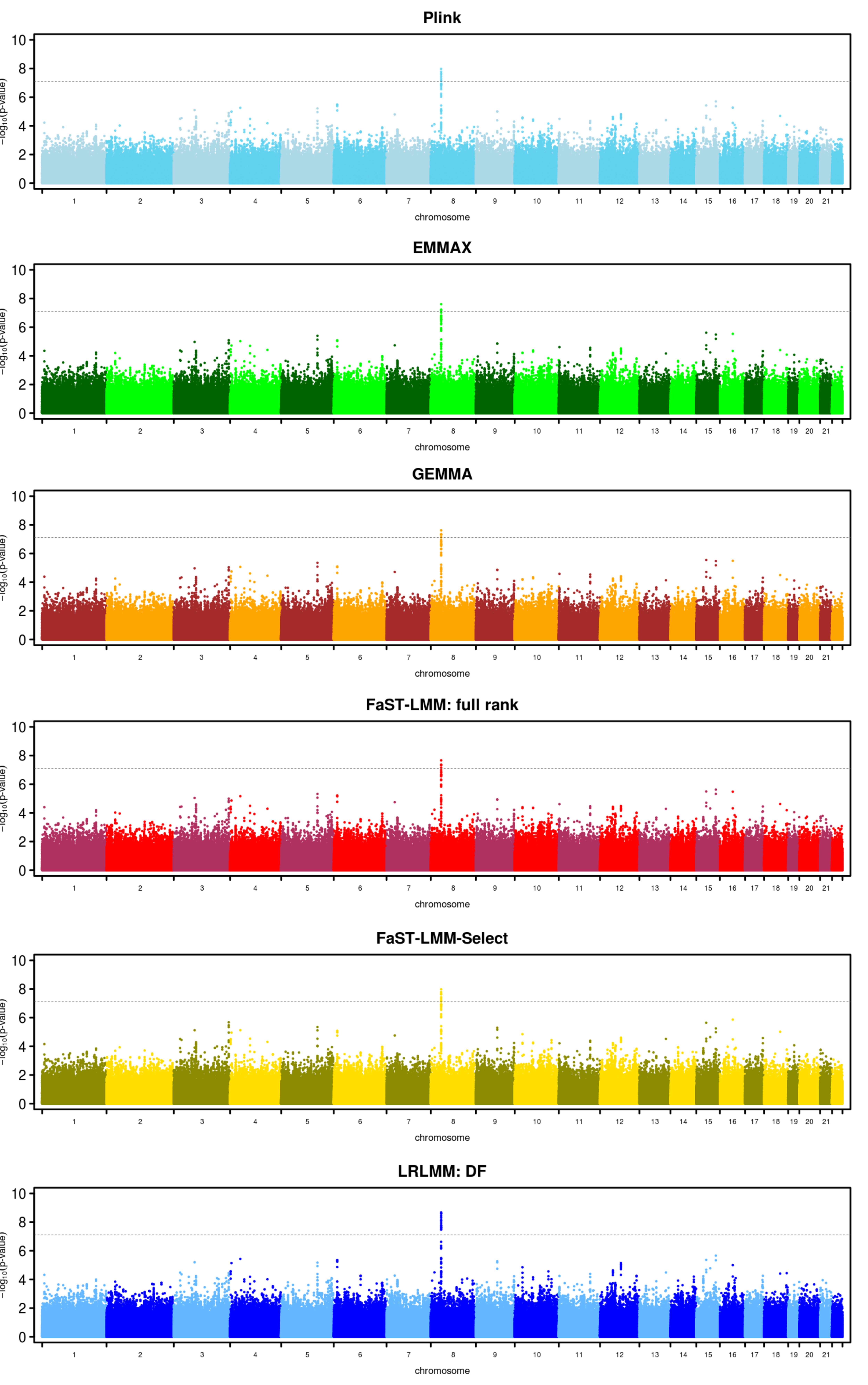

Supplement: Figure S10 — Manhattan plots for HDL cholesterol in Europeans from the Multi-Ethnic Study of Atherosclerosis (MESA). Results shown using Plink, EMMAX, GEMMA, FaST-LMM: full rank, FaST-LMM-SELECT and our low rank linear mixed model sorting by degrees of freedom from fitting each principal component individually (LRLMM-DF). (TIFF) [file pone.0075707.s010.tiff]
