# Supplementary material for: Correcting for Population Structure and Kinship Using the Linear Mixed Model: Theory and Extensions
Source: PLoS One. 2013 Oct 28;8(10):e75707. doi: 10.1371/journal.pone.0075707 (PMC3810480; doi:10.1371/journal.pone.0075707)
Supplement: File S1 — Eective degrees of freedom. (PDF) [file pone.0075707.s011.pdf]

Effective degrees of freedom

Show that $tr(\mathbf{H}) = \sum_i \frac{s_i^2}{s_i^2+\delta}$, letting $\delta = \frac{\sigma_e^2}{\sigma_a^2}$ and s_i^2 be the ith eigen-value of $\mathbf{K}$. The derivation follows from standard properties of matrix algebra and orthonormal matrices in the eigen-decomposition.

$$
\begin{aligned}
\mathbf{H} &= \mathbf{K}(\mathbf{K}+\mathbf{I}\delta)^{-1} \\
&= \mathbf{K}(\mathbf{U}\mathbf{S}^2\mathbf{U}^T + \mathbf{U}\mathbf{U}^T\delta)^{-1} \\
&= \mathbf{K}\left[\mathbf{U}(\mathbf{S}^2+\mathbf{I}\delta)\mathbf{U}^T\right]^{-1} \\
&= \mathbf{K}\mathbf{U}(\mathbf{S}^2+\mathbf{I}\delta)^{-1}\mathbf{U}^T \\
&= \mathbf{U}\mathbf{S}^2\mathbf{U}^T\mathbf{U}(\mathbf{S}^2+\mathbf{I}\delta)^{-1}\mathbf{U}^T \\
&= \mathbf{U}\mathbf{S}^2(\mathbf{S}^2+\mathbf{I}\delta)^{-1}\mathbf{U}^T
\end{aligned}
$$

$$
\begin{aligned}
tr(\mathbf{H}) &= tr[\mathbf{U}\mathbf{S}^2(\mathbf{S}^2+\mathbf{I}\delta)^{-1}\mathbf{U}^T] \\
&= tr[\mathbf{S}^2(\mathbf{S}^2+\mathbf{I}\delta)^{-1}\mathbf{U}^T\mathbf{U}] \\
&= tr[\mathbf{S}^2(\mathbf{S}^2+\mathbf{I}\delta)^{-1}] \\
&= \sum_i \frac{s_i^2}{s_i^2+\delta}
\end{aligned}
$$

We note that evaluating df_e for the low rank linear mixed model has the same form since no assumption of positivity was made for the values of s_i^2.
